# Volar Plate Repair for Chronic Injury

**DOI:** 10.1016/j.jhsg.2025.100776

**Published:** 2025-07-23

**Authors:** Michael Buldo-Licciardi, Marshall L. Balk, Robert J. Goitz

**Affiliations:** ∗ Department of Orthopedic Surgery, University of Pittsburgh, Pittsburgh, PA; † Hand and Shoulder Center, Wexford, PA

**Keywords:** Chronic, Finger, Repair, Tendon, Volar

## Abstract

**Purpose:**

The aim of this study was to assess the long-term outcomes of volar plate repair for chronic injury.

**Methods:**

Patients who underwent volar plate repair for chronic instability more than 6 months following the initial injury were included. A minimum follow-up of 2 years from time of surgery was required. Outcome measures included range of motion, the upper extremity *Quick*DASH (Disabilities of the Arm, Shoulder, and Hand) score, return to work, return to sport, and plain radiographs.

**Results:**

Ten patients were included. The mean time from injury to repair was 9 years, ranging from 10 months to 30 years. The digits involved included one thumb, four ring fingers, and five small fingers. Nine reported being extremely satisfied, and one reported being satisfied with their outcome at final follow-up. Nine of ten reported pain as their initial symptom, and none reported pain at final follow-up. Prior to surgery, all patients had proximal interphalangeal hyperextension ranging from 15° to 60°, three of which were classified as swan neck deformities. At final follow-up, nine patients had extension ranging from 0° to 3°. In addition, one patient had a hyperextension of 25°, although this patient had a subsequent injury. All had full flexion of their proximal interphalangeal joint at final follow-up. The three subjects who reported occupational impairment prior to surgery had no functional limitations following surgery. Two subjects whose injuries led to sport limitations reported returning to their preinjury level of sport.

**Conclusions:**

Volar plate repair for chronic injury resulted in successful outcomes based on satisfaction, *Quick*DASH score, physical examination, and radiographic images. These benefits were noted in a repairs performed decades after injury.

**Type of study/level of evidence:**

Therapeutic IV.

The volar plate (VP) consists of fibrocartilage tissue originating from the proximal phalanx neck and anchors into the distal phalanx base with lateral attachments to the accessory collateral ligaments.^1^ Its function is to reinforce the joint capsule, enhance joint stability, and limit hyperextension.^2^ Forced hyperextension of the proximal interphalangeal joint (PIP) causes volar plate avulsion from the base of the middle phalanx.^3^ Most injuries are generally tolerated well and heal uneventfully. However, some result in chronic hyperextension instability of the PIP joint which may necessitate surgical correction.^4^

Initial trauma to the VP is commonly dismissed as it is considered a stable injury with no long-term functional limitations. However, in some instances, persistent hyperextension and instability can occur with chronic VP incompetence.^5^ This can result in painful snapping of the lateral bands or even swan neck deformity.^6^ Many clinicians view a chronically injured VP unsuitable for repair and perform a flexor digitorum superficialis (FDS) tenodesis.^7^ Studies have explored the FDS technique for persistent VP injury.^8^, ^9^, ^10^ However, few have investigated VP repair.

The purpose of this multicentered study is to assess long-term outcomes of VP repair in patients with chronic injury. Our hypothesis is that a damaged VP is amenable to repair and that correction leads to long-term stable outcome with high patient satisfaction.

## Materials and Methods

After receiving institutional review board approval, we retrospectively reviewed patients who were diagnosed with chronic VP injuries and were treated with repair at two medical centers between January 1, 2000, and December 31, 2018. Patients were identified through a query of the institutions’ electronic medical record systems using the key words: chronic, PIP, plate, repair, and volar. Once an initial list was obtained, patients were individually screened for eligibility. Inclusion criteria included (1) a VP injury for at least 6 months and (2) a minimum of 2 years from time of surgery. Exclusion criteria included (1) concomitant procedures at time of surgery and (2) revision procedures.

Outcomes were based on patient satisfaction, change in finger pain, the validated upper extremity *Quick*DASH (Disabilities of the Arm, Shoulder, and Hand) score, physical examination and radiographic imaging.^11^ Satisfaction was rated on a five-point scale from extremely dissatisfied to extremely satisfied. Pain was rated on a 10-point scale, with 0 being no pain at all and 10 being the worst pain ever went through. The upper extremity *Quick*DASH score comprises of 11 questions related to daily activity, four supplementary questions related to return to sport, and four supplementary questions related to return to work. The scale is out of 100%, with 0% being no difficulty with any activity. Physical examinations were performed either in person or on a video visit and focused on PIP and distal interphalangeal (DIP) flexion and extension. PIP and DIP range of motions ranged from 10° of extension to 90° of flexion and were measured using a goniometer. Before surgery and final radiographic images were compared for signs of progressive degenerative disease.

The surgical technique is described as follows. The patient was prepared and draped in routine sterile fashion. A Bruner incision was made volarly over the PIP joint. The skin was elevated, and the neurovascular structures were retracted both radially and ulnarly. An incision was then made between the A2 and the A4 pulley. The FDS and the flexor digitorum profundus were retracted, the ruptured VP was identified and partially released both medially and laterally to advance it. The insertion site was curetted. Two anchors with 3-0 braided suture were used to repair the VP back to the insertion site in approximately 20° to 30° of flexion. The lateral edge of the VP both radially and ulnarly were repaired to the accessory collateral ligaments. The remainder of the pulley was placed between the A2 and A4 pulley deep to the flexor tendons. The skin was repaired with 4-0 nylon. A 20° dorsal block splint was maintained for 6 weeks.

## Results

Twenty-six patients who underwent VP repair for chronic instability were identified, and fifteen fulfilled the study criteria. Thirteen could be contacted, and ten agreed to participate in the study (Table 1). Four of the ten agreed to radiographic imaging.

**Table 1:** Demographic Characteristics of the Study Cohort

No.	Age (y)	Sex (Male/Female)	Dominant Hand	Injured Finger	Time From Injury to Repair	Occupation	Physical Activity/Sport	Follow-up (y)	Preop PIP Hyperextension (°)	Postop PIP Extension (°)
1	55	M	Right	L little	25 y	Construction	-	11	40	–5
2	51	M	Right	L little	30 y	Sales	Volleyball	2.5	60 (swan neck)	–5
3	22	M	Right	R ring	2 y	Grad school	Football	2.5	30	0
4	26	M	Right	R little	3 y	Computer engineer	Soccer	3	25	25
5	76	F	Right	R ring	8 y	Retired	-	15	40	–20
6	46	M	Right	R little	10 mo	Police officer	-	2	20	0
7	62	M	Left	R small	3 y	Mechanic	-	4	50	0
8	58	M	Left	L ring	20 y	Pianist, guitarist	-	6	20	3
9	28	M	Right	R ring	2 y	Service technician	Basketball	5	15	0
10	56	M	Right	R middle	15 y	Retired	Baseball	10	40	0

At final follow-up, the mean patient age was 48 years old, ranging from 22 to 76 years old. The mean time from injury to repair was 9 years, ranging from 10 months to 30 years. The digits involved included one thumb, four ring fingers, and five small fingers. Prior to surgery all patients had PIP hyperextension ranging from 15° to 60°, three of which were classified as swan neck deformities.

### Patient-reported outcomes

Nine patients reported being extremely satisfied with their surgery, and one reported being satisfied. Nine patients’ chief complaint prior to surgery was pain and one was function. None of the 10 patients reported pain at final follow-up. Average *Quick*DASH score was 3.3% (0% to 15.9%). Three patients had difficulty with their sport before surgery including one football player, one baseball player, and one volleyball player. The football and baseball players returned to their preinjury level. The volleyball player returned at a higher level compared to when they were injured, but not their preinjury level. Three other patients had occupational disabilities prior to surgery. A mechanic had difficulty turning a socket wrench, a police officer had trouble holding a gun, and an engineer had difficulty typing on the computer. All three returned to their preinjury work function after surgery.

### Physical examination

**Figure 1:**
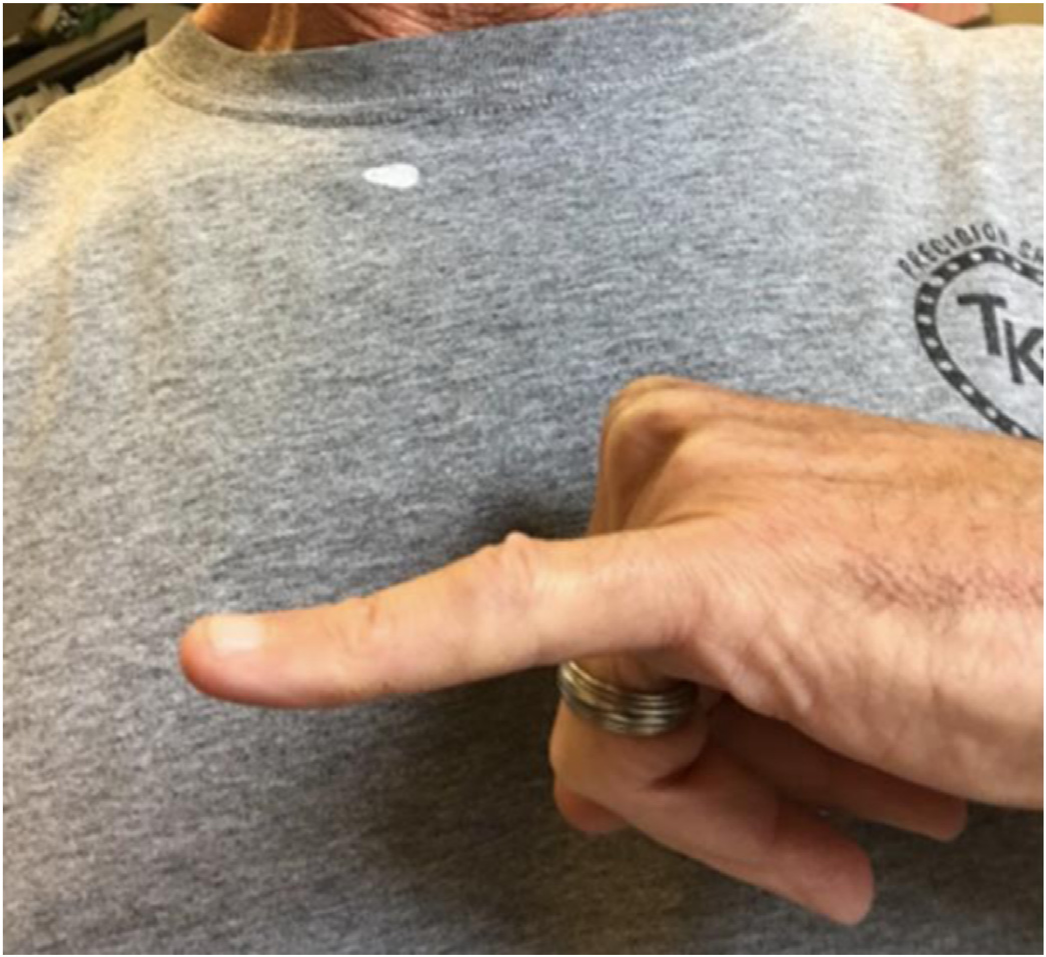
Eleven years after surgery in patient with 25 years between injury and repair.

**Figure 2:**
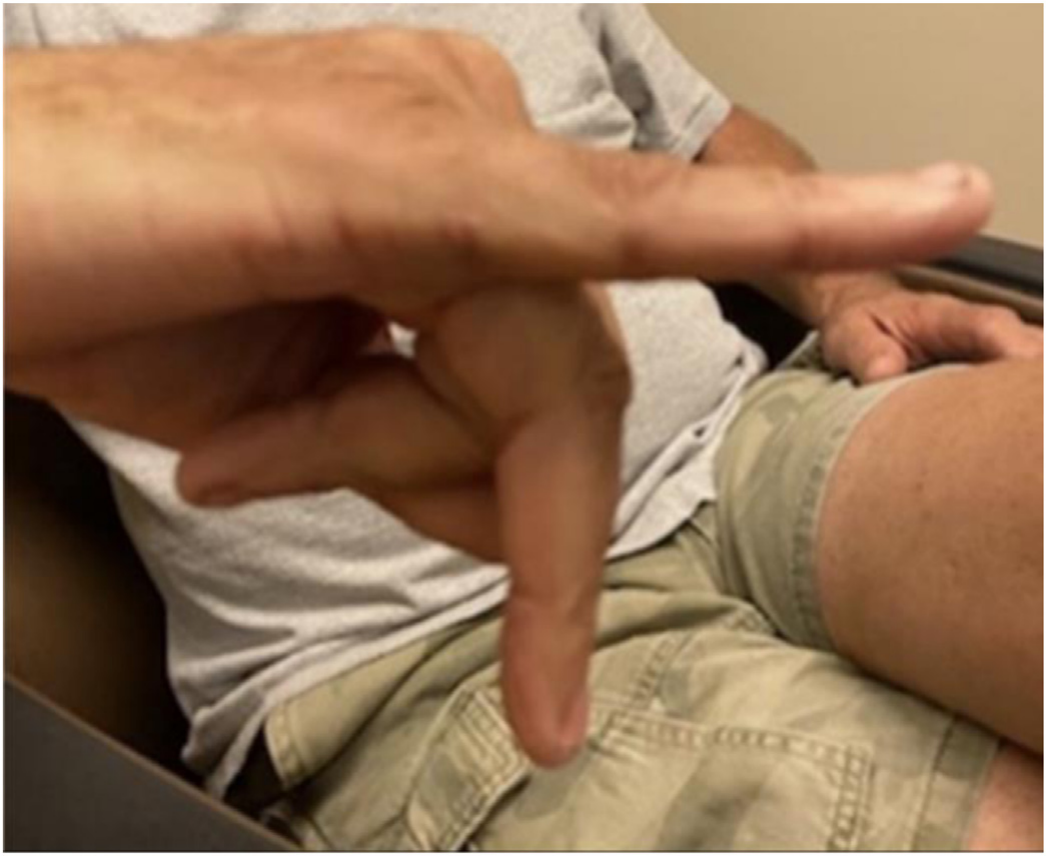
Four years after surgery in patient with 3 years between injury and repair.

**Figure 3:**
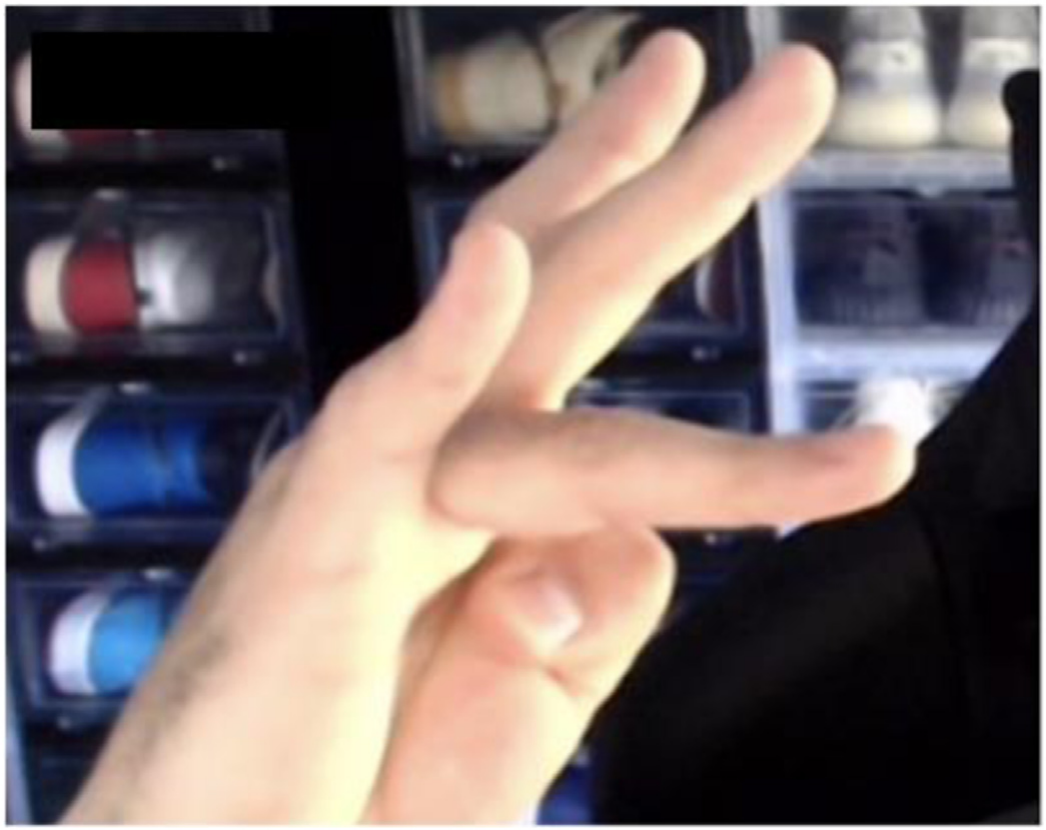
Five years after surgery in patient with 2 years between injury and repair.

Prior to surgery, the average hyperextension was 34° ranging from 15° to 60°. At final follow-up, six patients had extension ranging from 0° to 3°. Three patients had a flexion contracture at −5°, −5°, and −20°, separately. One patient had a subsequent injury jamming the finger on the back of a bus seat upon the bus stopping quickly and had a hyperextension of 25°. All patients had full flexion and extension of their DIP joint. The Figure 1, Figure 2, Figure 3 depict extension of three patients at final follow-up.

### Radiographic findings

**Figure 4:**
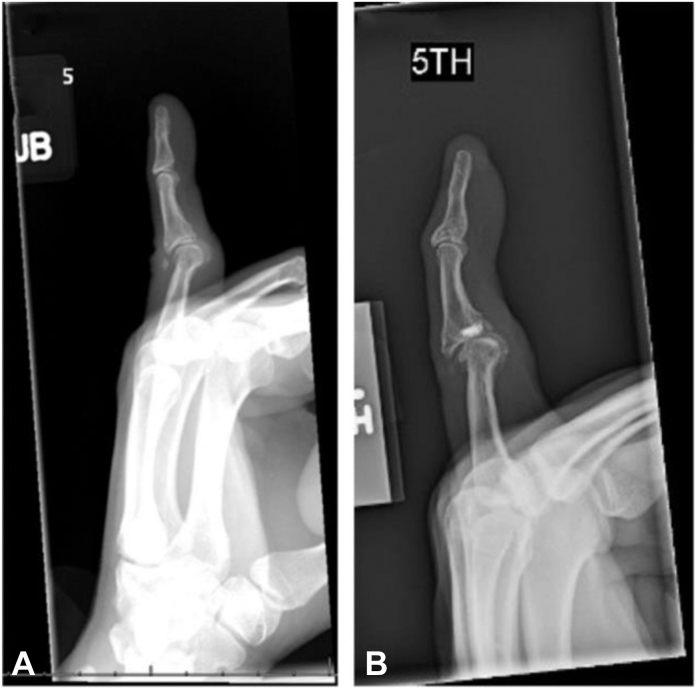
**A** Preoperative radiograph of a 21-year-old patient with a chronic VP injury. **B** A 2.5-year postoperative radiograph of a repaired VP 30 years after injury.

**Figure 5:**
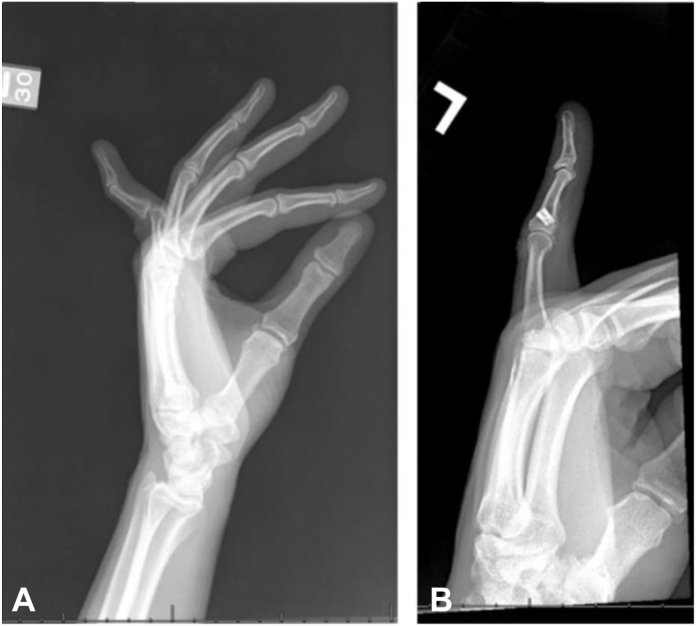
**A** Preoperative radiograph of a 30-year-old patient with a chronic VP injury. **B** An 11-year postoperative radiograph of a repaired VP 25 years after injury.

Four patients received radiographic images that showed no considerable progression of joint degeneration. Preoperative image and a postoperative image 2.5 years after surgery for a patient who underwent repair 30 years after injury (Fig. 4). Preoperative image and a postoperative image 11 years after surgery for a patient who underwent repair 25 years after injury (Fig. 5).

## Discussion

This study presents outcomes of ten patients with chronic VP injuries who underwent primary repair 10 months–30 years after initial injury. At final follow-up, nine were extremely satisfied, and one was satisfied with the procedure. Nine of ten reported pain as their chief complaint before surgery. None of the 10 patients reported pain at final follow-up. Following surgery, two patients who had difficulty with sport returned to their preinjury level, and one returned at an improved level. All three patients with difficulty at work returned to their preinjury level. Radiographic findings on four patients found no considerable progression of osteoarthritis.

To our knowledge this study includes the longest time from injury to repair case recorded for chronic VP injury, namely, 30 years. This patient had complete resolution of function and symptoms and a nearly complete return to preinjury level of sport. Prior to surgery, his finger would abruptly hyperextend during volleyball and require manual reduction, which has not occurred since his procedure.

Many physicians view a chronic VP injury as being unsuitable for repair and thus correct the defect via a FDS tenodesis. In 2003, Catalano et al^12^ reported excellent or good results in 10 of 12 patients who underwent FDS transfer for chronic injury using the original Littler tenodesis technique. In 2007, Onishi et al^13^ presented the first FDS transfer using two suture anchors. Since then there have been numerous variations of the FDS transfer technique studied. 8, 9, 10^,^^14^^,^^15^ Most recently, in 2018, Rocchi et al^7^ found that of the 13 patients who underwent FDS transfer using a minibone anchor, seven had complete resolution of pain, and instability and six had incomplete symptom resolution.

There have been few case series studying the outcome of direct repair. In 2006, Wollstein et al^16^ reported on 52 patients whose time from injury repair was 3 months to 6 years. The study found notable improvement in arc, flexion, and extension. At final follow-up the nonsurgical finger had considerably higher grip strength compared to the operated finger.^16^ In 2010, Melone et al^17^ reported outcomes on 25 patients whose times from injury to repair were 2 months to 23 years. The study found alleviation of pain in all patients and excellent/good results in 23 of the 25 patients. The average range of motion was 2° to 84° and radiographs in seven patients showed mild degenerative changes.^17^ In 2015, Kaneshiro et al^18^ reported all seven patients (range from injury to repair was 3 months to 17 years) being satisfied with their outcome following repair for chronic injury.^18^ In 2016, Lee et al^19^ found no pain following surgery and an average PIP ROM of 92.5° to 30° (flexion-extension) in 6 patients (range from injury to repair was 4 weeks to 3 months). Our results are comparable with the previous literature, finding improvement in pain, a low average *Quick*DASH score, high ROM, and minimal degenerative changes at final follow-up in patients up to 30 years from injury to repair.

We note several limitations of our analysis. As with all case series, our study lacked a comparison group; thus, we are unable to draw any conclusions about how outcomes of repair compare to the available alternatives. Patient-reported outcomes could not be obtained before surgery for the patients in this study. Perioperative experiences and outcome satisfaction could all influence patients’ willingness to participate in the study, thus introducing a selection bias.

## Conflicts of Interest

No benefits in any form have been received or will be received related directly to this article.
